# Supplementary material for: Modified musculofascial lengthening technique for submuscular ulnar nerve transposition in cubital tunnel syndrome
Source: PLoS One. 2025 Jan 29;20(1):e0318303. doi: 10.1371/journal.pone.0318303 (PMC11778715; doi:10.1371/journal.pone.0318303)
Supplement: Attachment — Submitted filename: Respone to Reviewers.docx [file pone.0318303.s003.docx]

**Response to Reviewers**

**We would like to thank you for the opportunity to resubmit our revised manuscript. We would also like to express our gratitude to the reviewers for their positive feedback and helpful comments. The manuscript has benefited immensely from these insightful suggestions. We have improved the article based on the critiques and suggestions. Please, see below our comments to the critiques and our revised manuscript. We have provided point-by-point responses to each suggestion below. The changes are also highlighted in the revised manuscript.**

Reviewer #2: The manuscript has undergone a thorough revision, and all questions and suggestions have been addressed well.
I would recommend, if possible, that the authors include the results of tests for ulnar nerve intrinsic muscle function before and after surgery to demonstrate any recovery of ulnar intrinsic muscle function, such as Froment's test, the Crossed Finger test, and Wartenberg's sign. If this is not feasible, the authors should mention in the limitations section the absence of complete data from intrinsic muscle testing to demonstrate postoperative improvement.

🡪 Page 9 (Line 203-4): Thank you for your insightful suggestion. In our study, we did not perform specific physical examinations to test the function of the intrinsic muscles innervated by the ulnar nerve, such as Froment's sign, Crossed Finger test, and Wartenberg's sign. We acknowledge that this is a limitation of our study. We have now included a statement in the limitations section to address this point:
